# Deficits in hippocampal neurogenesis in obesity-dependent and -independent type-2 diabetes mellitus mouse models

**DOI:** 10.1038/s41598-020-73401-9

**Published:** 2020-10-01

**Authors:** Jacqueline A. Bonds, Aashutosh Shetti, Terilyn K. L. Stephen, Marcelo G. Bonini, Richard D. Minshall, Orly Lazarov

**Affiliations:** 1 Department of Anatomy and Cell Biology, University of Illinois at Chicago, 578 CME (M/C 512), 808 South Wood Street, Chicago, IL 60612 USA; 2 Division of Hematology/Oncology, Department of Medicine, Feinberg School of Medicine and The Robert H. Lurie Comprehensive Cancer Center, Feinberg School of Medicine of Northwestern University, Chicago, IL 60612 USA; 3 Department of Anesthesiology, University of Illinois at Chicago, Chicago, IL 60612 USA; 4 Department of Pharmacology, University of Illinois at Chicago, Chicago, IL 60612 USA

**Keywords:** Alzheimer's disease, Adult neurogenesis

## Abstract

Hippocampal neurogenesis plays an important role in learning and memory function throughout life. Declines in this process have been observed in both aging and Alzheimer’s disease (AD). Type 2 Diabetes mellitus (T2DM) is a disorder characterized by insulin resistance and impaired glucose metabolism. T2DM often results in cognitive decline in adults, and significantly increases the risk of AD development. The pathways underlying T2DM-induced cognitive deficits are not known. Some studies suggest that alterations in hippocampal neurogenesis may contribute to cognitive deterioration, however, the fate of neurogenesis in these studies is highly controversial. To address this problem, we utilized two models of T2DM: (1) obesity-independent *MKR* transgenic mice expressing a mutated form of the human insulin-like growth factor 1 receptor (IGF-1R) in skeletal muscle, and (2) Obesity-dependent db/db mice harboring a mutation in the leptin receptor. Our results show that both models of T2DM display compromised hippocampal neurogenesis. We show that the number of new neurons in the hippocampus of these mice is reduced. Clone formation capacity of neural progenitor cells isolated from the db/db mice is deficient. Expression of insulin receptor and epidermal growth factor receptor was reduced in hippocampal neurospheres isolated from db/db mice. Results from this study warrant further investigation into the mechanisms underlying decreased neurogenesis in T2DM and its link to the cognitive decline observed in this disorder.

## Introduction

Type-2 diabetes mellitus (T2DM) is a chronic disease characterized by a state of persistent hyperglycemia and insulin resistance. T2DM is associated with a number of pathologies, including cardiovascular disease and neurodegeneration. Particularly alarming is the significant risk of developing cognitive deficits and Alzheimer’s disease (AD). Insulin resistance and T2DM increase the risk of AD by nearly two-fold compared to non-diabetics^1^. The combined overall relative risk for dementia (including clinical diagnoses of both AD and vascular dementia) is 73% higher in people with T2DM than in those without^2,3^. In turn, AD patients experience brain insulin resistance and hyperinsulinemia^4,5^. The increased risk of AD in type 2 diabetes mellitus (T2DM) patients suggests that systemic processes in T2DM lead to brain pathology and cognitive impairments. Brain MRI suggested increased frequency of cerebral atrophy in T2DM compared to age- matched individuals without T2DM^6^. Similarly, a significant and progressive reduction in brain weight was observed in db/db mice^7,8^. However, the cognitive mechanisms failing in T2DM are not fully understood. In mouse models of T2DM, cognitive deficits were associated mostly with impairments in the hippocampus^9^. For example, changes in synaptic plasticity were observed in hippocampal slices of Streptozotocin-diabetic rats ^10^. Specifically, long-term potentiation (LTP) was impaired in the dentate gyrus (DG), CA3 and CA1 regions, while long-term depression was facilitated in CA1^10^.

Hippocampal neurogenesis plays important roles in the regulation of hippocampal function, learning and memory^11^. In addition, impaired hippocampal neurogenesis is linked to AD^12–14^. Several reports suggest that hippocampal neurogenesis is altered in mouse models of T2DM. However, the information is often contradictory or inconclusive. For example, the proliferation of bromodeoxyuridine positive (BrdU +) cells was increased in the subventricular zone (SVZ) and subgranular zone (SGZ) of the DG of Goto-Kakizaki (GK) rat, while their survival significantly decreased. These alterations took place in parallel to increased blood glucose level^15^. However, the identity of these BrdU + cells was not specified. Stranahan and colleagues (2008) report that the numbers of BrdU + cells and BrdU + Tuj1 + are reduced in the SGZ of db/db mice compared to wild type^16^. In contrast, another study reports increased number of BrdU + or DCX + cells in the SVZ, SGZ and cortex of db/db mice. Here too, the identity of BrdU + cells was not studied. In addition, it is not clear whether the number of cells was adjusted to brain volume, given the progressive atrophy^8^. In the context of obesity-related T2DM, a high-fat diet was reported to impair the number of proliferating cells in the SGZ of the hippocampus in male, but not female rats^17^. In contrast, a recent study shows that high-fat diet compromises neurogenesis in C57BL/6 J females but not in males^18^.

Interestingly, several studies suggest that metformin, a drug used to manage T2DM, has pro-neurogenic characteristics^19^. For example, Wang and colleagues observed that metformin enhances the differentiation of embryonic cortical NPCs, as well as of adult-born NPCs in the SVZ and the SGZ in mice^20^. Chronic administration of metformin facilitates cell proliferation and neuronal differentiation and inhibits diabetes-related neuroinflammation in the brains of DIO mice^21^. Treatment of the neonatal mice with metformin following hypoxia-induced brain injury facilitated neurogenesis and led to functional recovery^22^. This effect has been proposed to be mediated via insulin receptor substrate 1^21^, while another study suggests that the effect on self-renewal and proliferation occurs via the p53 family member and transcription factor TAp73, and it promotes neuronal differentiation of these cells by activating the 5′ adenosine monophosphate-activated protein kinase/protein kinase C/transcriptional coactivator CREB-binding protein pathway AMPK-aPKC-CBP pathway^20,23^. Another study suggests that GLP-1 mimetic Exendin-4, which is used for the treatment of T2DM, enhances hippocampal neurogenesis in three mouse models of T2DM: *ob/ob*, *db/db* and high‐fat‐diet‐fed mice^24^. Taken together, these studies suggest that while there is a possible connection between hippocampal neurogenesis and cognitive deficits in T2DM, the fate of hippocampal neurogenesis in T2DM is yet to be established.

To address the current controversy, we analyzed two mouse models of T2DM that may represent different disease mechanisms: obesity-dependent db/db mice, expressing a spontaneous mutation in the leptin receptor (*Lepr*^*db*^), and obesity-independent MKR mice, expressing the human insulin-like growth factor I receptor (*IGF-1R*) gene containing the K1003R mutation driven by the murine creatine kinase, muscle (*Ckm*) promoter. The extent of neural stem cells and new neurons in the hippocampus of these mice was examined using neurogenic proxies. Consistently, in both models we observed deficits in the number of new neurons as well as deficits in neural progenitor cell proliferation.

## Results

### Alterations in the proliferative and differentiation capacity of neural progenitor cells in db/db model of T2DM

**Figure 1:**
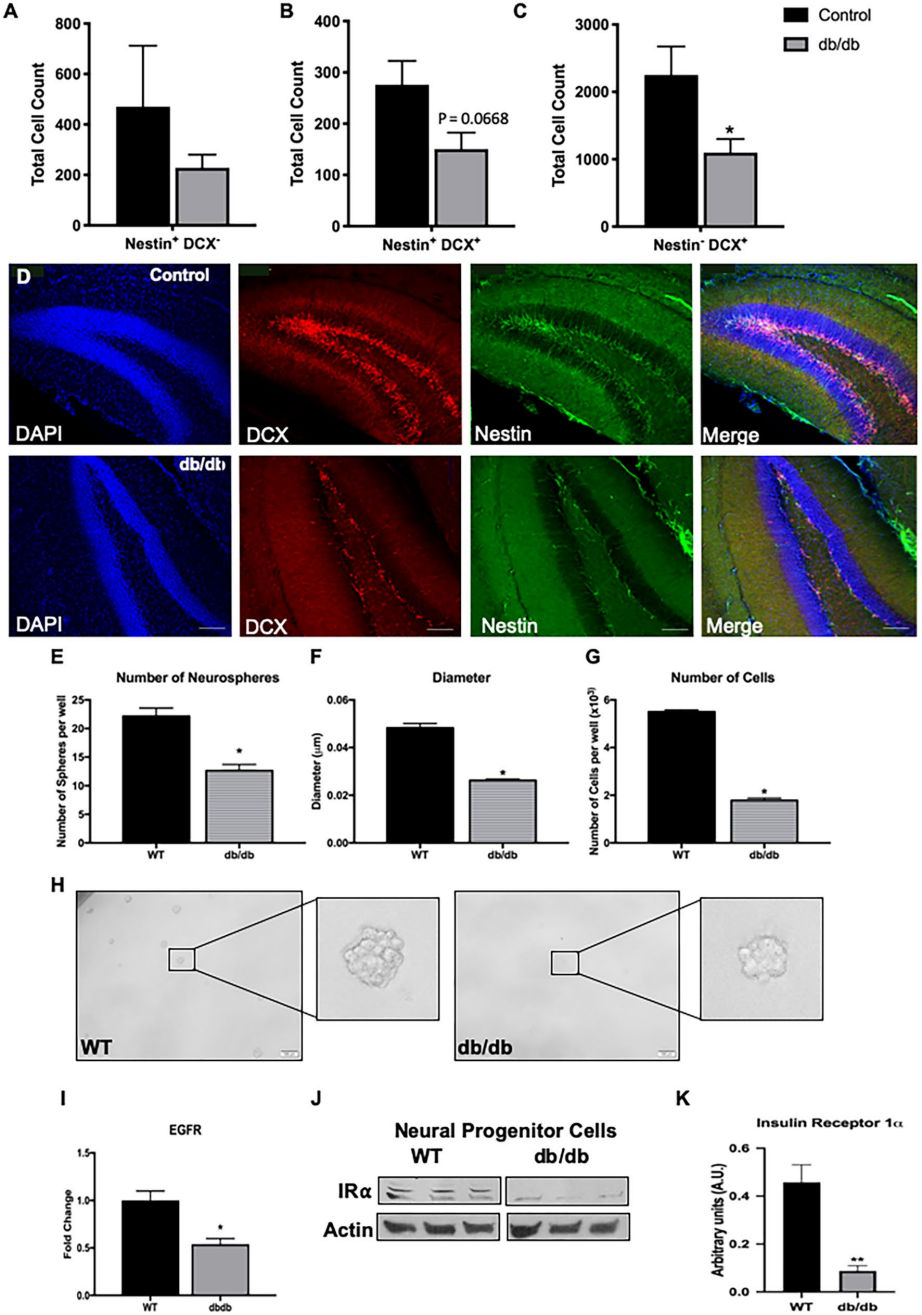
Reduced number of new neurons in the SGZ of db/db mice. (**A**–**C**) Quantification of the number of neural progenitor cells (Nestin + DCX−, **A**), neuroblasts (Nestin + DCX + , **B**) and immature neurons (Nestin− DCX + , **C**) in the subgranular zone of the dentate gyrus of db/db and wild type mice using unbiased stereology. The number of neural progenitor cells was similar between genotypes. There was a trending decrease in the number of proliferating neuroblasts (*P* = 0.0668, N = 4). A significant decrease was observed in the number of immature neurons (*P < *0.05*) in the db/db mice (Unpaired t-test, 12 weeks of age, N = 4). (**D**) Representative images of wild type and db/db brain sections immunolabeled with antibodies raised against doublecortin (DCX) or nestin and counterstained with DAPI. Scale bar = 75 μm. (**E**–**G**) Clonogenic assay of primary neural progenitor cells isolated from the dentate gyrus of db/db and wild type mice. Analysis of the number (**E**) and diameter (**F**) of neurospheres formed, as well as the total number of cells (**G**) show a significant decrease in all parameters in the db/db compared to wild type. (**H**) Representative images of neurospheres cultured from wild type and db/db mice (N = 3, unpaired t-test, *P < 0.05). Inserts show high power images of a single neurosphere. (**I**) Real time PCR analysis of mRNA expression of epidermal growth factor receptor (EGFR) in primary neurospheres shows a significant decrease in the db/db genotype (N = 3, unpaired t-test, *P < 0.05). (**J,K)** Western blot analysis (**J**) and semi-quantification using image J (**K**) of the expression of insulin receptor 1α (IR1α) in protein lysate of neurospheres isolated from db/db and wild type mice. Expression of IR1α is severely compromised in the db/db genotype (N = 3, unpaired t-test, *P < 0.05).

To determine the extent of hippocampal neurogenesis in T2DM, we first quantified the number of cells expressing nestin and/or doublecortin (DCX) in the subgranular zone (SGZ) in brain sections of 12-week-old male db/db mice using unbiased stereology (Fig. 1A–D). We observed that the number of neural progenitor cells (NPCs, nestin + DCX−) was similar in db/db and wild type mice (Fig. 1A). The number of neuroblasts (nestin + DCX +) was reduced in db/db, albeit not statistically significant (*P* = 0.0668, N = 4, Fig. 1B,D). Interestingly, the number of immature neurons (Nestin−DCX +) was significantly lower in db/db mice compared to wild type (*P* = 0.0490, N = 4, Fig. 1C,D), suggesting less new neurons in db/db mice, particularly as age and disease progress. To further examine this, NPCs were isolated from the DG of db/db and wild type mice and grown in culture to form neurospheres. Next, neurospheres were singly dissociated and subjected to a clone-forming assay. Quantification revealed that the number and size of neurospheres and the total number of cells were reduced in the db/db cultures compared to wild type neurospheres (N = 3, Fig. 1E–H). This may suggest that proliferation of NPCs derived from the db/db mice is reduced compared to wild type mouse NPCs. Another possibility is that the survival of NPCs or neuroblasts derived from db/db mice is compromised. To start to explore these possibilities, we examined the expression levels of epidermal growth factor receptor (EGFR) and insulin receptor 1α (IR-1α), respectively. EGF is a critical proliferation factor of NPCs. Insulin plays a major role in neuronal differentiation and is likely to be compromised in the T2DM brain. In the healthy brain, IR-1α is highly abundant in the hippocampus and in NPCs and regulates hippocampus-dependent learning and memory^25^. The expression of EGFR and IR-1α were examined in RNA extracts and protein lysates, respectively, of neurosphere cultures derived from the brains of db/db and wild type mice. We observed a significant reduction in the expression of EGFR in db/db NPCs compared to wild type mouse NPCs (Fig. 1I, N = 3). In addition, we observed a marked reduction in the expression level of IR-1α in db/db NPCs compared to wild type NPCs (Fig. 1J,K; N = 3). Taken together, these results suggest impairments in the proliferation and differentiation of new neurons in the hippocampus of db/db mice at 12 weeks of age.

### Impairments in hippocampal neurogenesis in the MKR mouse recapitulate impairments in the db/db

**Figure 2:**
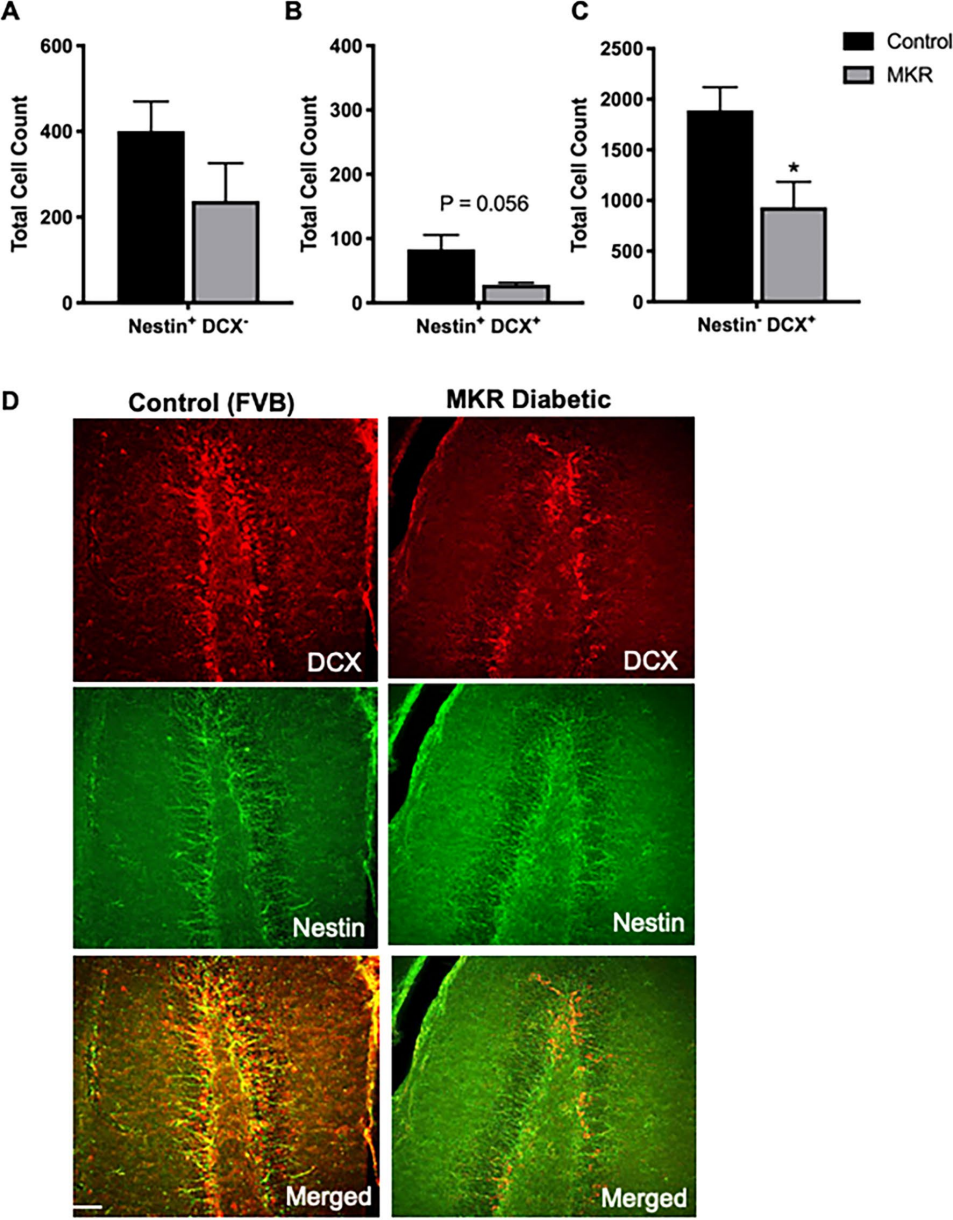
Reduced number of new neurons in the subgranular zone is recapitulated in MKR mice. (**A**–**C**) Stereological analysis of neural progenitor cells (Nestin + DCX−, **A**), neuroblasts (Nestin + DCX + , **B**) and immature neurons (Nestin−DCX + , **C**) in the subgranular zone of the dentate gyrus of MKR and wild type FVB mice. The number of neural progenitor cells was similar between genotypes. A trending decrease was observed in proliferating neuroblasts (Nestin^+^ DCX^+^; *P* = 0.056) and a significant decrease of immature neuroblasts (Nestin^−^ DCX^+^) in MKR mice compared to FVB control (N = 6, Unpaired t-test, *P < 0.05). (**D**) Representative confocal images of brain sections of MKR and wild type mice stained with antibodies raised against Nestin and DCX. Scale bar = 75 μm.

Given that there are several mechanisms leading to T2DM, we chose to investigate an obesity-independent model (the MKR mouse) to determine if the impairments observed in the db/db mouse represent the state of neurogenesis in T2DM. Similar to findings in the *db/db* brains, results show that levels of NPCs (Nestin + DCX−) were comparable in the hippocampi of MKR and wild type mice (Fig. 2A, N = 6). Interestingly, we observed a nearly significant decrease in the number of proliferating neuroblasts (Nestin + DCX +) in the hippocampi of MKR compared to wild type, which could indicate deficits in neuroblast proliferation or neuronal maturation (Fig. 2B, N = 6). In agreement with reduced number of neuroblasts, a significant decrease was observed in the number of immature neurons (Nestin−DCX +) in the hippocampus of MKR mice (Fig. 2C,D, N = 6).

### Differential alterations in neural stem cell population in hippocampus of db/db and MKR mice

**Figure 3:**
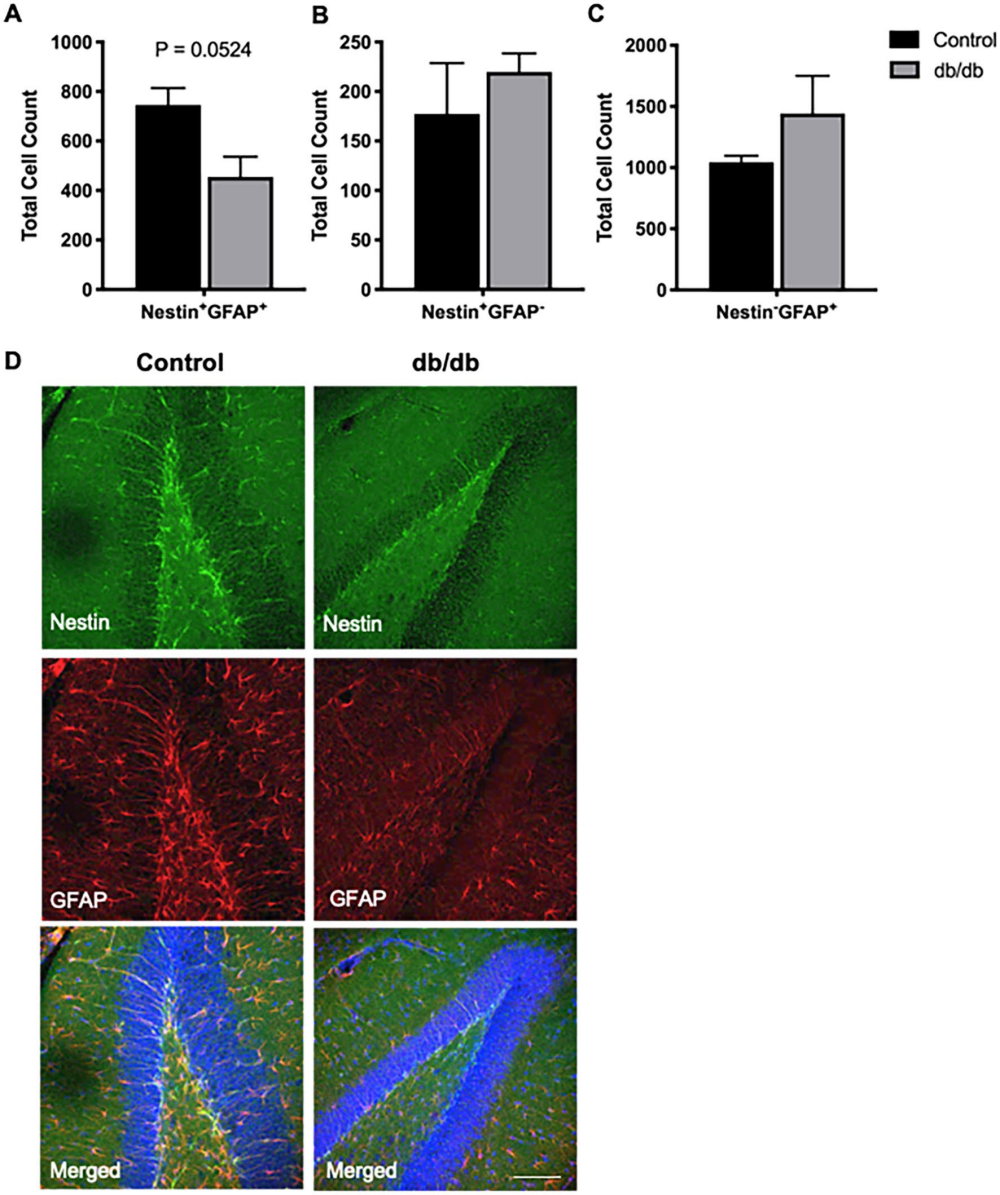
Alterations in neural stem cell number in db/db mice. (**A**–**C**) Quantitative analysis of the number of neural stem cells (Nestin + GFAP + , **A**), neural progenitor cells (Nestin + GFAP−, **B**) and mature astrocytes (Nestin− GFAP + , **C**) in the subgranular zone of the dentate gyrus in db/db and wild type mice shows a trending decrease in the number of neural stem cells in db/db mice (*P* = 0.0524). The number of neural progenitors or mature astrocytes was similar in both genotypes (N = 3, 12 weeks of age, Unpaired t-test). (**D**) Representative confocal images of neural stem cells in the dentate gyrus in brain sections of db/db and wild type mice. Scale bar = 75 μ.

To determine if impairments in neurogenesis involve alterations in the neural stem cell (NSC) pool, we next quantified the number of Nestin + GFAP + in the hippocampus of db/db and wild type mice. Intriguingly, there was a reduction in the number of Nestin + GFAP + cells in the hippocampi of db/db mice compared to the wild type, albeit not statistically significant (*P* = 0.0524, N = 3, Fig. 3A,D). There was no change in the level of Nestin + GFAP−, which designates several populations of NPCs at different stages of development (Fig. 3B, N = 3). Interestingly, we further observed an increase in the number of mature astrocytes (Nestin−GFAP +) in the db/db compared to the wild type mice, however it was not significant (Fig. 3C, N = 3).

**Figure 4:**
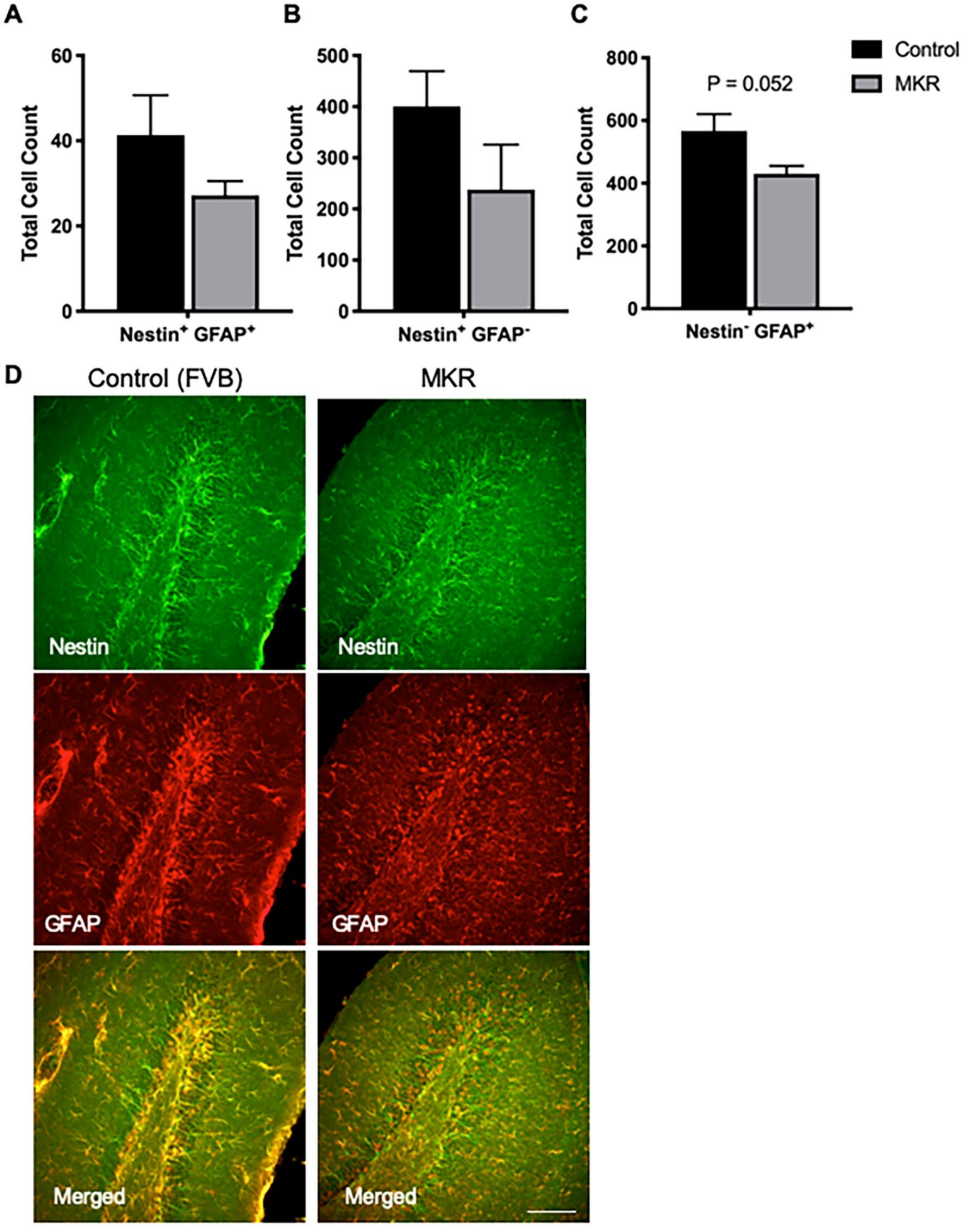
MKR mice display trending loss of mature astrocytes in the subgranular zone of the dentate gyrus. (**A**–**C**) The number of neural stem cell (Nestin + GFAP + , **A**) and neural progenitor cells (Nestin + GFAP−, **B**) was similar in db/db and wild type mice at 12 weeks of age, as determined by unbiased stereology. However, MKR mice display a trending decrease in the number of mature astrocytes (Nestin− GFAP + , *P* = 0.052), (N = 6, Unpaired t-test). (**D**) Representative confocal images of Nestin + and GFAP + cells in the subgranular zone of the dentate gyrus of MKR and wild type mice. Scale bar = 75 μm.

Examination of these populations in the MKR mice revealed that the number of NSCs (Nestin + GFAP + , N = 6, Fig. 4A) in the hippocampus was lower in db/db compared to the wild type mice, albeit not statistically significant (*P* = 0.202). No change was observed in the total number of NPCs (Nestin + GFAP−, N = 6, Fig. 4B). In contrast to the db/db, there was a trending decrease in the number of mature astrocytes (*P* = 0.052, N = 6, Fig. 4C). However, alterations in the number of mature astrocytes did not reach significance in neither genotype, and the lack of consistency warrants further investigation.

In summary, based on the results of this study, we conclude that hippocampal neurogenesis is deficient in both T2DM mouse models used here, manifested by reduced number of new neurons. We further observed reduced levels of EGFR and IR in NPCs derived from the hippocampi of db/db mice, which may underlie some of these deficits.

## Discussion

This study addresses discrepancies in the literature concerning the fate of neurogenesis in T2DM mouse models. These discrepancies are likely the result of lack of use of appropriate neurogenic proxies, and inadequate or lack of quantification. Nevertheless, determining the fate of hippocampal neurogenesis in the brains of T2DM patients and mouse models is critical for understanding the mechanism of cognitive deterioration. Recent intriguing studies show that hippocampal neurogenesis is impaired in mild cognitive impairment and AD, as well as in mouse models of AD^12,13^. This may imply that dysfunction of hippocampal neurogenesis in T2DM may contribute to the development of AD. To this end, in this study we show that the number of new neurons is reduced in the SGZ of the hippocampus of two mouse models of T2DM, suggesting that less new neurons are available for the recruitment and incorporation in the granular layer of the DG. This is significant because it may lead to hippocampal dysfunction and cognitive deficits, and may provide a putative mechanism underlying the substantial portion of T2DM patients who deteriorate cognitively. Observing reduced number of new neurons in the hippocampus of two different mouse models of T2DM validates this result as physiologically—relevant and excludes the possibility that it is a transgene- related off-target effect. Notably, MKR mice express a mutated form of the human insulin-like growth factor 1 receptor (IGF-1R) in *skeletal muscle* solely, and yet, pathology develops in the brain and in neural progenitor cells and new neurons specifically and recapitulates the impairments observed in the db/db mouse.

Second, our observations showed trending reductions in the numbers of Nestin + DCX + cells in the SGZ of both the db/db and MKR mice. This result was strengthened by the observed reduction in cell proliferation in neurospheres isolated from db/db mice. Nevertheless, it should be noted that the population of cells positive for Nestin + DCX + , as well as the population of cells encompassing neurospheres in vitro, each includes several subpopulations that proliferate at different rates. In addition, a difference in clone-formation capacity in culture could be due to mechanisms other than proliferation, for example, cell adhesion. Further investigation is needed in order to clarify the specific mechanisms underlying the reduced numbers of proliferating NPCs and neuroblasts in these mouse models.

Third, we observed reduced expression of IR-1α in NPCs derived from the db/db mouse. We cannot exclude the possibility that this may be due to the mutation in leptin receptor expressed in the db/db mice. Specifically, previous studies suggest that both insulin and leptin circulate at levels that vary in proportion to body fat stores and both enter the CNS in proportion to their plasma level, where they act on their respective receptors^26^. Deficiency of either hormone or their respective receptors in the CNS results in insulin resistance^27,28^. In light of the tight functional association, it is expected that the mutation in leptin receptor would affect insulin receptor expression and signaling. Thus, levels of IR-1α in NPCs should be validated in other mouse models of T2DM.

Another mechanism by which insulin resistance or deprivation can compromise neurogenesis is via the Wnt3-NeuroD1 pathway. Previous studies have shown that Wnt3 promotes neuronal differentiation by the activation of the transcription factor NeuroD1, which in turn, induces the expression of insulin^29–31^. Notably, deficiency of NeuroD1 causes malformation of the DG and sever diabetes^32–34^. Lastly, it should be noted that insulin, IGF-1, and IGF-2 also play a role in oligodendrocyte differentiation and neuronal survival^32–34^. Thus, dysfunctional insulin signaling would have major implications for neurogenesis and gliogenesis.

Fourth, in addition to IR-1α, we observed that levels of EGFR are reduced in NPCs derived from the db/db mouse. EGF is a critical regulator of NPC proliferation. Interestingly, EGFR can be compromised directly or indirectly by lack of leptin receptor signaling. Evidently, a leptin-EGFR-Notch 1 axis has been described under pathological conditions^35^. Interestingly, a recent study described EGF-like metabolic hormones that bind EGFR as critical modulators of neural activity, coupling insulin secretion to the nutritional status in Drosophila^36^. Future studies should address the mechanism by which deficits in EGFR and IR-1α in NPCs play a role in altered hippocampal neurogenesis in T2DM.

Fifth, inconsistencies were found between db/db and MKR as to the number of NSCs and mature astrocytes as compared to their respective wild type strains. This may be either genotype related or a representation of differential effects of diabetic mechanisms on the neurogenic population in the two mouse models. These observations warrant further investigations addressing the effect of distinct molecular signaling mechanisms on hippocampal neurogenesis. In that regard, it should be noted that this study examined levels of hippocampal neurogenesis in male mice. Sex differences in levels of neurogenesis have been reported in T2DM related mouse models^17,18^. Thus, future studies should include a comparative analysis of neurogenesis in both sexes.

In summary, the results of this study suggest that hippocampal neurogenesis is deficient in mouse models of T2DM. Deficient neurogenesis may contribute to the development of cognitive impairment in T2DM patients. Understanding the mechanism underlying defective neurogenesis may help alleviate T2DM-linked memory impairments.

## Materials and methods

### Mice

All animal experiments were approved by the University of Illinois at Chicago Institutional Animal Care and Use Committee, ACC Protocol # 17–123 (Lazarov) and # 16–204 (Minshall). All experiments were performed in accordance with institutional guidelines and regulations.

All mice used in this study were obtained from Jackson Laboratories. Twelve week old db/db and wild-type control & 4–5-month-old male MKR diabetic and FVB control mice were used in this study. FVB/NJ (stock # 001800), MKR (FVB-Tg(Ckm-IGF*K1003R)1Dlr/J, stock #016618), C57B6 (stock #000664), db/db (BKS.Cg-Dock7^m^ + / + Lepr^db^/J, stock #000642).

### Immunohistochemistry

Following perfusion with cold PBS, brains were post-fixed in 4% PFA for 24 h. Brains were rinsed three times with PBS and transferred to 30% sucrose for 24 h. Tissue sections were obtained on a freezing microtome (Leica) where they were serially sectioned at 50 µm. Sections were washed three times at room temperature in 1 × TBS for 5 min. Tissue was then blocked in blocking buffer (1 × TBS, 0.3 M glycine, 5% normal donkey serum, 0.25% Triton X-100) for 2 h at room temperature and then incubated in primary antibodies, diluted in blocking buffer, for 72 h at 4 °C. Primary antibodies: Nestin (EMD Millipore, Mouse, Cat# MAB353), DCX (Santa Cruz Biotechnologies, Goat, Cat# sc-8066) GFAP (Dako, Rat, Cat# Z0334). Tissue was then washed three times at room temperature in 1 × TBS for 5 min and incubated in secondary antibodies, diluted in blocking buffer, for 2 h at room temperature. Secondary antibodies obtained from Jackson-Immuno (Biotin anti-Mouse, Cat# 715-065-150; Streptavidin Cy2, Cat# 016-220-084; Donkey anti-Rat Cy3, Cat# 712-166-150; Donkey anti-Goat Cy3, Cat# 705-165-147). Tissue was then washed three times at room temperature in 1 × TBS for 5 min and incubated in DAPI (0.5 µg/mL; ThermoFisher Scientific #D1306) for 5 min at room temperature and then mounted and stored at 4 °C.

### Unbiased stereology

Cell counts were performed using design-based stereology (StereoInvestigator, MBF Biosciences). For the analysis, every sixth section of brain tissue was quantified using the optical fractionator workflow of StereoInvestigator. Regions of interest were traced under 5 × magnification with counting performed under 63 × magnification with a counting frame of 225 µm × 145 µm and sampling grid of 100 µm × 100 µm with 12.5 µm top and bottom guard zones.

### Primary neurosphere culture

#### Isolation

8–12 weeks old mice were euthanized via isoflurane overdose and brains dissected into HBSS stored on ice followed by dissection of the hippocampus. Tissue was minced with a sterile scalpel and transferred to 3 mL warm media (DMEM/F-12, 20 mM KCl, 2 μg/mL heparin, 1% penicillin–streptomycin, 20% B27 supplement, 10% N2 supplement). After tissue was settled, media was removed, and tissue was dissociated with 0.1% trypsin–EDTA and incubated with agitation at 37 °C for 7 min. Trypsin inhibitor (139 μg/mL plus 10 μg/mL DNase I in HBSS^-/-^) was added and samples spun at 700 rpm for 5 min. Tissue pellet was resuspended in 1 mL warm media supplemented with 20 ng/mL EGF and 10 ng/mL bFGF and pipetted 20 × with a P1000 pipet and cultured at 37 °C, 5% CO_2_ and passaged every 7 days.

#### Passaging

Media is collected and spun at 1000 rpm for 5 min. Neurospheres are dissociated with 0.05% trypsin–EDTA and incubated in a 37 °C water bath for 7 min. Trypsin inhibitor was added, and cells spun at 1000 rpm for 5 min. Cells are counted and plated at 10,000 cells/cm^2^.

### Western blot analysis

Cells were collected and homogenized in lysis buffer (150 mM Na_2_CO_3_, 1 mM EDTA, pH 8) containing protease inhibitor cocktail (1:100, Sigma) and sonicated 3 × for 15 s. Protein was quantified via microplate BCA method, normalized to equal concentrations, and boiled at 95 °C for 10 min with 4 × sample buffer (0.35 M Tris–HCl, pH 6.8, 30% glycerol, 5% β-Mercaptoethanol, 10% SDS, bromophenol blue). Protein was separated via SDS-PAGE. Membranes were blocked for 30 min in 5% non-fat dry milk in PBST (PBS + 0.1% Tween-20), then incubated in primary antibody overnight at 4 °C with shaking. Membranes were placed in appropriate HRP secondary antibody in blocking buffer for 1 h at room temperature with shaking. Levels of protein were quantified using densiometric measurements from ImageJ Software (La Jolla, CA). All protein levels were normalized to levels of actin unless otherwise noted.

### Quantitative reverse transcription polymerase chain reaction

RNA was extracted from cultured cells using the ISOLATE II RNA Mini Kit (Bioline USA, Inc, Taunton, MA, USA) according to manufacturer’s instructions and was quantified using a Nanodrop and stored at − 80 °C until used. cDNA synthesis was performed using the SuperScript™ III First-Strand Synthesis SuperMix (ThermoFisher Scientific) according to manufacturer’s instructions starting with 1 µg total RNA per sample and using oligo(dT) priming and stored at − 20 °C until used. qPCR was performed using SensiFAST™ SYBR^®^ and Fluorescein Kit (Bioline USA, Inc, Taunton, MA, USA). Each reaction was done using 1 µL of starting cDNA. Relative expression was calculated using the ΔΔCt method using RPLP0 as the housekeeping gene. Primer sequences for EGFR (Gene ID 13649) were Forward (5′- GCCATCTGGGCCAAAGATACC-3′) and Reverse (5′- GTCTTCGCATGAATAGGCCAAT-3′).

### Statistical analyses

All statistical analyses were performed in GraphPad Prism (Version 7.0a; GraphPad Software Inc). Data shown are ± SEM and a probability of less than 0.05 was considered significant, using unpaired two-tailed t-test. Western blot analyses were done by densitometry and compared using the unpaired two-tailed t-test normalized to actin levels (noted as arbitrary units or A.U.).
